# Time course of plasma gelsolin concentrations during severe sepsis in critically ill surgical patients

**DOI:** 10.1186/cc6988

**Published:** 2008-08-17

**Authors:** HaiHong Wang, BaoLi Cheng, QiXing Chen, ShuiJing Wu, Chen Lv, GuoHao Xie, Yue Jin, XiangMing Fang

**Affiliations:** 1 Department of Anesthesiology, the First Affiliated Hospital, School of Medicine, Zhejiang University, QingChun Road, Hangzhou 310003, PR China; 2 Key Laboratory of Multiple Organ Transplantation, Ministry of Public Health, the First Affiliated Hospital, School of Medicine, Zhejiang University, QingChun Road, Hangzhou 310003, PR China

## Abstract

**Introduction:**

Gelsolin is an actin-binding plasma protein that is part of an 'actin-scavenging' system. Studies suggest that plasma gelsolin may play a crucial role in the pathophysiology of sepsis. Little is known about the course of plasma gelsolin levels over time in patients with severe sepsis. The aim of the study was to investigate plasma gelsolin levels in severe septic patients and to determine whether these levels predict the severity or clinical outcome of severe sepsis.

**Methods:**

Ninety-one patients who were diagnosed with severe sepsis at admission to a surgical intensive care unit were enrolled, and admission plasma gelsolin levels were recorded. Plasma gelsolin levels were recorded daily in 23 of these patients. Daily plasma gelsolin levels were recorded in an additional 15 nonseptic critically ill patients. Fifteen volunteers served as healthy control individuals. Plasma gelsolin levels were measured using an enzyme-linked immunosorbent assay. Concentrations of IL-6, IL-10 and tumour necrosis factor (TNF)-α were also measured on intensive care unit admission.

**Results:**

The admission gelsolin levels were significantly decreased in severe sepsis (20.6 ± 11.7 mg/l) compared with nonseptic critically ill patients (52.3 ± 20.3 mg/l; *P *< 0.001) and healthy control individuals (126.8 ± 32.0 mg/l; *P *< 0.001). Severe septic patients had increased IL-6 levels compared with nonseptic critically ill patients (20.0 ± 10.7 pg/ml versus 11.4 ± 13.9 pg/ml; *P *= 0.048), whereas no significant difference in IL-10 or TNF-α levels was observed (IL-10: 97.9 ± 181.5 pg/ml versus 47.4 ± 91.5 pg/ml, respectively [*P *= 0.425]; TNF-α: 14.2 ± 13.9 pg/ml versus 6.9 ± 5.3 pg/ml, respectively; *P *= 0.132). Survivors of severe sepsis exhibited substantial recovery of their depressed plasma gelsolin levels, whereas gelsolin levels in nonsurvivors remained at or below their depleted admission levels.

**Conclusion:**

Plasma gelsolin may be a valuable marker for severe sepsis. Recovery of depleted plasma gelsolin levels correlated with clinical improvement. The prognostic role of plasma gelsolin in critical illness requires further investigation in a large cohort.

## Introduction

Gelsolin, a protein of 82 to 84 kDa, is a member of gelsolin protein superfamily, which exists in a cytoplasmic as well as an excreted plasma isoform, and contains six homologous repeats termed gelsolin-like (G) domains [1-3]. Plasma gelsolin is the principal circulating protein able to sever and scavenge circulating filamentous actin [4-6], which may enhance some major components of proinflammatory cytokine production, impair the microcirculation and compromise multiple organs [7-10]. In animal models, plasma gelsolin appears to be beneficial, possibly by virtue of its ability to counteract the pathophysiological consequences of actin release during trauma, injury and infection [11-14].

In animal models of sepsis, depletion of plasma gelsolin correlates with elevated circulating levels of actin and gelsolin replacement modifies the cytokine profile and improves survival [14]. In humans the plasma gelsolin levels are markedly decreased in acute liver failure, myocardial infarction, septic shock, myonecrosis and allogeneic stem cell transplantation, and the degree of depletion correlates with the degree of organ dysfunction, as measured using disease-specific markers [15,16]. Soon after traumatic injury, plasma concentrations of gelsolin are significantly reduced compared with those in healthy individuals [17]. Admission plasma gelsolin levels in patients admitted with a variety of critical illness were associated with the development of acute respiratory distress syndrome and septic shock [15,17]. Lee and coworkers [18] serially measured plasma gelsolin levels in patients after surgery or trauma for 5 days and demonstrated that the decreased plasma gelsolin levels seen in such patients are stable over this period. However, that study did not assess the time course of gelsolin recovery or its correlation with clinical improvement.

We studied plasma gelsolin levels at the time of admission to the intensive care unit (ICU) in patients with severe sepsis. Additionally, we measured daily plasma gelsolin levels in critically ill patients admitted to a surgical ICU with severe sepsis and without sepsis. Plasma gelsolin levels were also measured in a cohort of healthy volunteers. Gelsolin levels were compared among these groups and changes in these levels were observed over time to determine whether these changes were associated with outcomes in patients with severe sepsis.

## Materials and methods

This study was performed in accordance with the ethical guidelines of the School of Medicine, Zhejiang University. The protocol was approval by the Institutional Review Board (Ethics Committee). Written informed consent was obtained from both patients and healthy volunteers. In cases in which consent was obtained from the relatives of patients who were unable to give it, consent was later obtained from patients who regained the ability to do so. Patients in this prospective observational study were cared for in a surgical ICU at a university hospital. Patients with severe sepsis were enrolled upon admission to the ICU, using the criteria of the American College of Chest Physician/Society of Critical Care Medicine Consensus Conference Committee [19]. Exclusion criteria were any of the following: lack of informed consent, age younger than 18 years, and pre-existing immunological or haematological diseases. In addition to demographic information, Acute Physiology and Chronic Health Evaluation II (APACHE II) [20] scores and Sequential Organ Failure Assessment (SOFA) [21] were recorded for all patients with severe sepsis. Deaths were defined as all-cause, in-hospital deaths. Fifteen nonseptic critically ill patients at the same ICU during the study period were enrolled randomly as the critically ill control group. In addition, 15 volunteers served as the healthy control group. All patients and volunteers were of Chinese Han origin.

Whole blood samples were obtained from 91 consecutive patients with severe sepsis from 1 June 2006 to 31 May 2007 within 24 hours after ICU admission. Among these patients, daily plasma gelsolin levels were measured in 23 consecutive patients. Day 0 was defined as the time of admission into the surgical ICU. Whole blood samples were also obtained daily from 15 nonseptic, critically ill patients at ICU admission and the following 5 consecutive days. A single whole blood sample was obtained from each of the 15 healthy volunteers. Whole blood samples (3 ml) were collected into EDTA-containing tubes. After being centrifuged at 2,500 *g *for 5 minutes, plasma was harvested and frozen at -80°C until analysis. Plasma gelsolin levels were measured using an enzyme-linked immunosorbent assay, in accordance with the manufacturer's instructions (CoTimes, Beijing, China). Admission plasma tumour necrosis factor (TNF)-α, IL-6, IL-10 and albumin levels were determined using enzyme-linked immunosorbent assay, in accordance with the manufacturer's instructions (R&D systems, Minneapolis, MN, USA) in both the severe sepsis group and the nonseptic critically ill group.

Normally distributed data are presented as mean ± standard deviation and compared using Student's *t*-test. Non-normally distributed data are presented as median and interquartile range, and compared using Mann-Whitney U-test. For consistency with previous studies, some variables with unknown/non-normal distribution (for instance, plasma level of TNF-α, IL-6, and so on) were presented as mean ± standard deviation. Noncontinuous variables are presented as percentages and were analyzed using χ^2 ^test or Fisher's exact test. A logistic regression was performed with the occurrence of severe sepsis as the dependent factor and admission plasma gelsolin, IL-6, IL-10, TNF-α, albumin, age and sex as independent factors. The Forward methodology was adopted in the regression process. A variable would enter the model with a *P *value under 0.05 and would be removed with a *P *value greater than 0.10. All statistical analysis was performed with SPSS 14.0 for Windows (SPSS, Chicago, IL, USA). P values under 0.05 (two-tailed) were considered statistically significant.

## Results

Of the 91 patients with severe sepsis enrolled in this study, 51 (56.0%) patients died in hospital. The underlying diseases associated with the development of severe sepsis were nosocomial pneumonia (n = 31), bowel obstruction (n = 14), severe acute pancreatitis (n = 14), intestinal or gastric perforation (n = 10), trauma (n = 10), infection of liver or biliary tree (n = 6) and others (n = 6). The demographic and clinical data for the patients are presented in Table 1. Among the 15 nonseptic critically ill control patients there were no deaths. No difference was observed in age and sex between nonseptic critically ill patients and patients with severe sepsis.

**Table 1:** Characteristics of patients with severe sepsis

Characteristic	Patients with severe sepsis		*P*
		
	Nonsurviving (n = 51)	Surviving (n = 40)	
Age (years)	68 (48 to 75)	57.5 (44.3 to 72.8)	0.15
Males (n [%])	29 (56.9)	27 (67.5)	0.386
Plasma concentration of gelsolin (mg/l; mean ± SE)	20.9 ± 11.2	20.2 ± 12.3	0.786
Plasma concentration of IL-6 (pg/ml; mean ± SE)	21.6 ± 11.6	19.0 ± 10.0	0.59
Plasma concentration of IL-10 (pg/ml; mean ± SE)	68.7 ± 56.0	127.3 ± 250.8	0.347
Plasma concentration of TNF-α (pg/ml; mean ± SE)	19.2 ± 17.7	9.2 ± 5.4	0.033
Plasma concentration of albumin (g/l; mean ± SE)	27.5 ± 6.2	25.1 ± 6.5	0.101
APACHE II (median [IQR])	24 (17.8 to 27.3)	17 (12.5 to 22)	<0.001
Severe SOFA (median [IQR])	9 (6 to 13.3)	6 (4 to 8)	0.002
Organs with acute dysfunction (n [%])			
Respiratory	48 (94.1%)	32 (80%)	0.054
Cardiovascular	20 (39.2%)	6 (%)	0.018
Renal	18 (35.3%)	6 (15%)	0.033
Haematologic	20 (39.2%)	14 (35%)	0.827
Central nervous system	35 (68.6%)	23 (57.5%)	0.283
Hepatic	24 (47.1%)	14 (35%)	0.288

APACHE, Acute Physiology and Chronic Health Evaluation II; IQR, interquartile range; SE, standard error; SOFA, Sequential Organ Failure Assessment; TNF, tumour necrosis factor.

**Figure 1:**
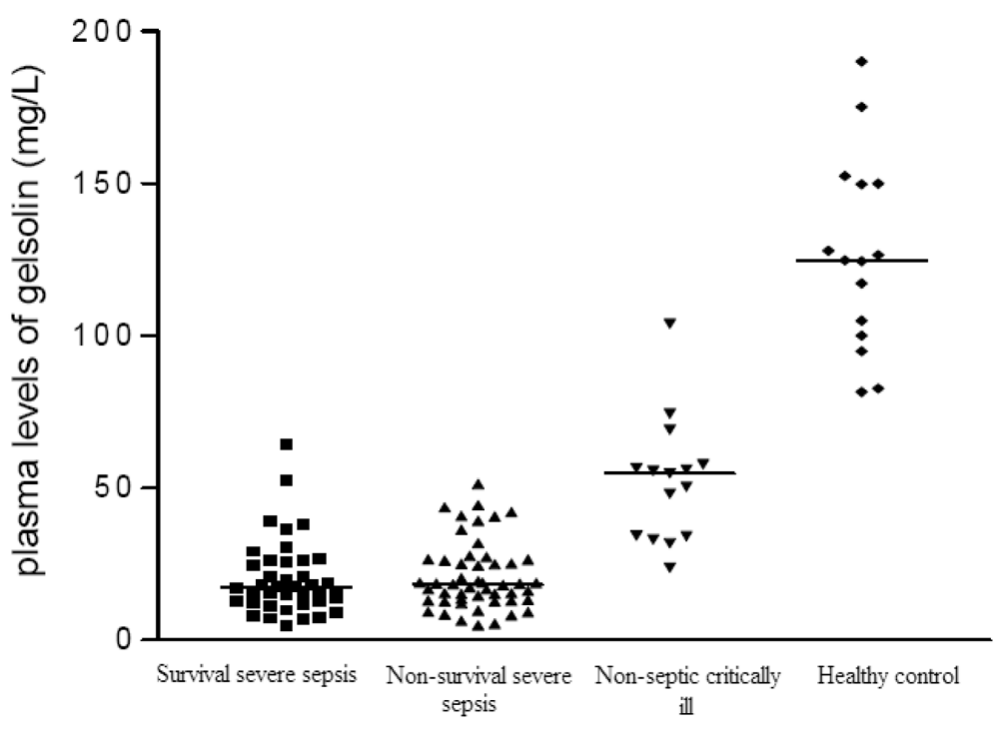
Admission plasma gelsolin levels. Presented is a comparision of the plasma gelsolin levels at the time of admission of survival severe sepsis, nonsurvival severe sepsis, nonseptic critically ill and healthy control.

As shown in Figure 1, the plasma gelsolin level at the time of admission in the severe sepsis group was 20.6 ± 11.7 mg/l, which was significantly lower than that of 15 nonseptic critically ill patients (52.3 ± 20.3 mg/l, *P *< 0.001). The admission levels of plasma gelsolin in both severe sepsis and nonseptic critically ill patients were significantly different from those of the 15 healthy control individuals (126.8 ± 32.0 mg/l; *P *< 0.001). Severe septic patients exhibited an increased IL-6 level compared with the nonseptic critically ill patients (20.0 ± 10.7 pg/ml versus 11.4 ± 13.9 pg/ml; *P *= 0.048), whereas no significant differences in IL-10 and TNF-α levels were observed between the two groups (IL-10: 97.9 ± 181.5 pg/ml versus 47.4 ± 91.5 pg/ml, respectively [*P *= 0.425]; TNF-α 14.2 ± 13.9 pg/ml versus 6.9 ± 5.3 pg/ml, respectively [*P *= 0.132]). Both the severe sepsis group and the nonseptic critically ill group had similar lowered plasma albumin levels (26.4 ± 6.4 g/l versus 29.2 ± 3.9 g/l; *P *= 0.071). Higher (≥ 25) APACHE II scores were associated with lower plasma gelsolin levels at ICU admission, as compared with lower (<25) scores (17.1 ± 9.1 mg/l versus 22.4 ± 14.4 mg/l; *P *= 0.044). In contrast, higher (≥ 8) admission SOFA scores were not associated with plasma gelsolin levels as compared with patients with lower (<8) score (21.5 ± 12.8 mg/l versus 19.7 ± 10.6 mg/l; *P *= 0.457).

Logistic regression revealed that among the seven candidate risk factors (admission plasma gelsolin, IL-6, IL-10, TNF-α, albumin, age and sex), admission plasma gelsolin was the only independent factor able to predict the occurrence of severe sepsis. However, there was no significant difference in the gelsolin levels between surviving and nonsurviving patients with severe sepsis (20.2 ± 12.3 mg/l versus 20.9 ± 11.2 mg/l; *P *= 0.786).

**Figure 2:**
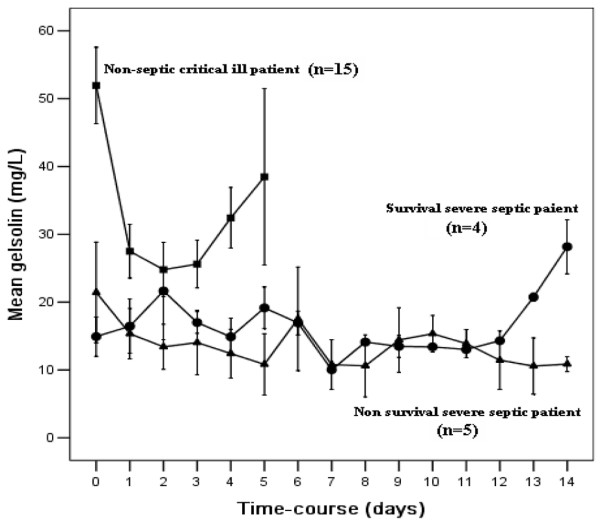
Time course of plasma gelsolin levels. Presented are the courses over time of plasma gelsolin levels in nonseptic critically ill patients, and patients with severe sepsis who survived and did not survive. Results are expressed as means ± standard error.

Twenty-three patients with severe sepsis and 15 nonseptic critically ill patients had daily plasma gelsolin levels measured consecutively after their ICU admission. Nine out of 23 consecutive septic patients who had been sampled for gelsolin levels daily stayed in the ICU for longer than 14 days. The time course of plasma gelsolin concentration in these nine septic patients is shown in Figure 2. Among survivors depressed plasma gelsolin levels appeared to recover after day 11, whereas plasma gelsolin levels remained low or even decreased further in the nonsurvivors with severe sepsis. For nonseptic critically ill patients, the depressed plasma gelsolin levels increased after day 3 of the surgical ICU stay, which was coincided with clinical improvement.

## Discussion

In the present study we found that admission plasma gelsolin levels were lower in patients with severe sepsis than in nonseptic critically ill ICU patients and healthy control individuals. Admission plasma gelsolin level was a independent risk factor that correlated with occurrence of severe sepsis, although it did not significantly differ between surviving and nonsurviving patients with severe sepsis. Recovery of plasma gelsolin levels was observed late in the course in survivors but not in nonsurvivors with severe sepsis.

In the study conducted by Lee and coworkers [14], depletion of plasma gelsolin in animal models of sepsis occurred 6 hours after a septic challenge with either endotoxin (lipopolysaccharide) or a polymicrobial challenge after caecal-ligation and puncture [14]. Several clinical studies [15,16] have observed that a low gelsolin level after an initial insult such as injury or inflammation reflected greater severity of disease and poorer outcomes. The depletion of plasma gelsolin soon after a septic challenge may result from exposure of the actin cytoskeleton, which occurs as part of cellular injury [22-24]. In turn, depletion of gelsolin could allow the formation of actin filaments, which would lead to further tissue injury and organ dysfunction [7-10,25]. In addition, plasma gelsolin binds bioactive inflammatory mediators including lipopolysaccharide [26], lysophosphatidic acid [27] and platelet-activating factor [28].

The present study revealed that plasma gelsolin levels measured at the time of ICU admission in patients with severe sepsis were lower than those in nonseptic critically ill patients and healthy control individuals. Although plasma albumin levels in both severe septic group and nonseptic critically ill group were below the normal value, there was no significant difference between the two groups. This indicates that the decrease in plasma gelsolin level was specific and not a simple consequence of systemic plasma protein loss or dilution. Combined with previous reports [14-18], this study suggests that early determination of plasma gelsolin level could facilitate early diagnosis of severe sepsis.

In contrast to the study conducted by Mounzer and coworkers [17], this study could not replicate a definite association between plasma gelsolin levels of admission and mortality. Mounzer and coworkers demonstrated that low plasma gelsolin levels at admission were associated with increased risk for adverse outcomes, including prolonged length of hospital stay and death, in patients who had undergone surgery or who had suffered trauma [17]. Possibly, the characteristics of patient population and the limited number of cases contributed to the conflicted results. Interestingly, Huang and colleagues [10] found that the plasma gelsolin level recovered at the time of clinical improvement. In the present study, among the 15 nonseptic critically ill patients that admitted to the surgical ICU for postoperative or post-traumatic observation, the decreased plasma gelsolin levels demonstrated a recovery after day 3. Furthermore, this study demonstrated that the depletion of plasma gelsolin recovered with clinical improvements in survivors of severe sepsis, whereas the gelsolin level in nonsurvivors remained low. This finding is consistent with the hypothesis proposed by Lee and coworkers [14], namely that plasma gelsolin can modify systemic inflammatory response and improve the outcome of sepsis via its binding and neutralizing inflammatory mediators during the course of sepsis. To our knowledge, this is the first study to examine the time course of plasma gelsolin changes and its correlation with clinical improvement in septic patients.

The limitations of the study were as follows. The number of patients with severe sepsis enrolled in the time course study is inadequate to allow definitive conclusions to be drawn, and the study does not unequivocally elucidate the role played by plasma gelsolin in sepsis or the association of plasma gelsolin with cytokines. Studies such as this one are important because the animal data suggest that repletion of low plasma gelsolin levels may be a useful adjuvant therapy, and it is critical that we detemine means to identify those patients who could potentially benefit from such therapy, if we are to optimize recombinant drug treatment in this setting. Further study is required to address these issues.

## Conclusion

This study suggests that plasma gelsolin levels are a valuable marker of severe sepsis in surgical ICUs. Admission plasma gelsolin levels correlated with severity of sepsis, whereas recovery of plasma gelsolin levels correlated with clinical improvement. The prognostic role played by plasma gelsolin level in critical illness needs to be further investigated in a large cohort.

## Key messages

• Admission plasma gelsolin levels in patients with severe sepsis were lower than those in nonseptic, critically ill ICU patients or healthy control individuals.

• Admission plasma gelsolin levels were associated with the occurrence of severe sepsis.

• Survivors of severe sepsis exhibited substantial recovery of their depressed plasma gelsolin levels, whereas the gelsolin levels in nonsurvivors remained at or below their depleted admission levels.

• Plasma gelsolin may be a valuable marker for severe sepsis.

## Abbreviations

APACHE: Acute Physiology and Chronic Health Evaluation; ICU: intensive care unit; IL: interleukin; SOFA: Sequential Organ Failure Assessment; TNF: tumour necrosis factor.

## Competing interests

The authors declare that they have no competing interests.

## Authors' contributions

HHW and BLC contributed equally to the manuscript. HHW, BLC, QXC and XMF contributed to the design of the study and drafted the manuscript. HHW, BLC, QHX, SJW and YJ enrolled the patients and participated in the laboratory work. HHW, BLC and CL contributed to data analysis and interpretation of the results. All authors read and approved the final manuscript.
